# Supplementary material for: Donor-derived CD8+CD122+ Tregs generated in mixed donor chimeric NOD mice delete autoreactive T cells
Source: bioRxiv. 2026 Mar 22:2026.03.20.712252. Preprint. [Version 1] doi: 10.64898/2026.03.20.712252 (PMC13015718; doi:10.64898/2026.03.20.712252)
Supplement: Supplement 1 — Fig S1. Gating hierarchy of murine CD8+CD122+ Tregs. Fig S2. Phenotypic characterization of CD8+CD122+ Tregs from NOR and NOD mice. Fig S3. Details of the conditioning regimen used for HSCT. Fig S4. Detection and characterization of IGRP-reactive CD8+ T cells. Fig S5. Donor CD8+CD122+ Tregs expand in the pancreas of chimeric NOD mice. Fig S6. Host CD25+ T cells isolated from NOD chimera prevent diabetes onset. Fig S7. Phenotype of bone marrow-derived d-CD8+CD122+ Tregs. Fig S8. Donor-derived CD8+CD122+ Tregs have reduced expression Scart1 and Scart2. Fig S9. Donor-derived CD8+CD122+ Tregs have higher cytotoxicity effects towards IGRP-reactive CD8+ T cells. Fig S10. Screening of CDR3 peptides using activation assay. Fig S11. MHC-I blockade abrogated CDR3 peptide-mediated activation of d-CD8+CD122+ Tregs. Fig S12. Proposed mechanism of IGRP-reactive CD8+ T cell killing by d-CD8+CD122+ Tregs in vitro. Fig S13. Phenotype of CD8+CD122+ Tregs in individuals with T1D. Fig S14. Representative flow cytometry plot showing overlapping phenotypic characteristics between CD8+CD122+ Tregs and CD8+CD158+ Tregs in human sample. [file media-1.pdf]

Supplementary Information

Donor-derived $CD8^+CD122^+$ Tregs generated in mixed donor chimeric NOD mice delete autoreactive T cells

Shiva Pathak[1,2,3], Cameron S. Bader[1,3], Bettina P. Iliopoulou[1,2,3], Shobha Regmi[4], Pin-I Chen[1,3], Biki Gupta[1], Xiangni Wu[1], Blake Mosher[1], Alexandra Wells[1], Levon Witherspoon[1], Kayla Jenkins[1], William Harper[1], Emily SooHoo[1], Abigail Twoy[1], Rizwan Ahmed[5], Suparna Dutt[6], Nadine Nagy[1,2,3], Kent P. Jensen[1,2,3], Garrison Fathman[5], Avnesh S. Thakor[7], Mark M. Davis[8,9], and Everett H. Meyer[1,2,3,*]

[1]Division of Blood and Marrow Transplantation and Cellular Therapy, Stanford University School of Medicine, Stanford, CA, USA

[2]Stanford Diabetes Research Center, Stanford University School of Medicine, Stanford, CA, USA

[3]Cellular Immune Tolerance Program, Stanford University School of Medicine, Stanford, CA, USA

[4]Department of Pediatric Gastroenterology, Stanford University School of Medicine, Stanford, CA, USA

[5]Department of Immunology and Rheumatology, Stanford University School of Medicine, Stanford, CA, USA

[6]Department of Radiation Oncology, Stanford University School of Medicine, Stanford, CA, USA

[7]Department of Radiology, Stanford University School of Medicine, Stanford, CA, USA

[8]Institute of Immunity, Transplantation and Infection, Stanford University School of Medicine, Stanford, CA, USA

[9]Department of Microbiology and Immunology, Stanford University School of Medicine, Stanford, CA, USA

***Address for correspondence**

Everett H. Meyer, MD, PhD
Associate Professor
Division of Blood and Marrow Transplantation and Cellular Therapy
Stanford University School of Medicine, Stanford, CA 94305, USA
Email: evmeyer@stanford.edu

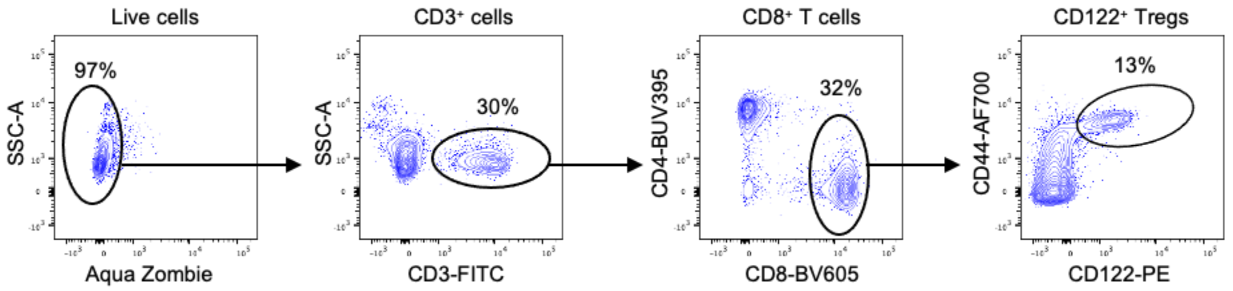

Fig S1. Gating hierarchy of murine CD8$^+$CD122$^+$ Tregs. Dead cells were excluded by staining with Zombie Aqua™ Fixable Viability Kit and CD3$^+$CD8$^+$CD44$^+$CD122$^+$ Tregs were gated from the live cells.

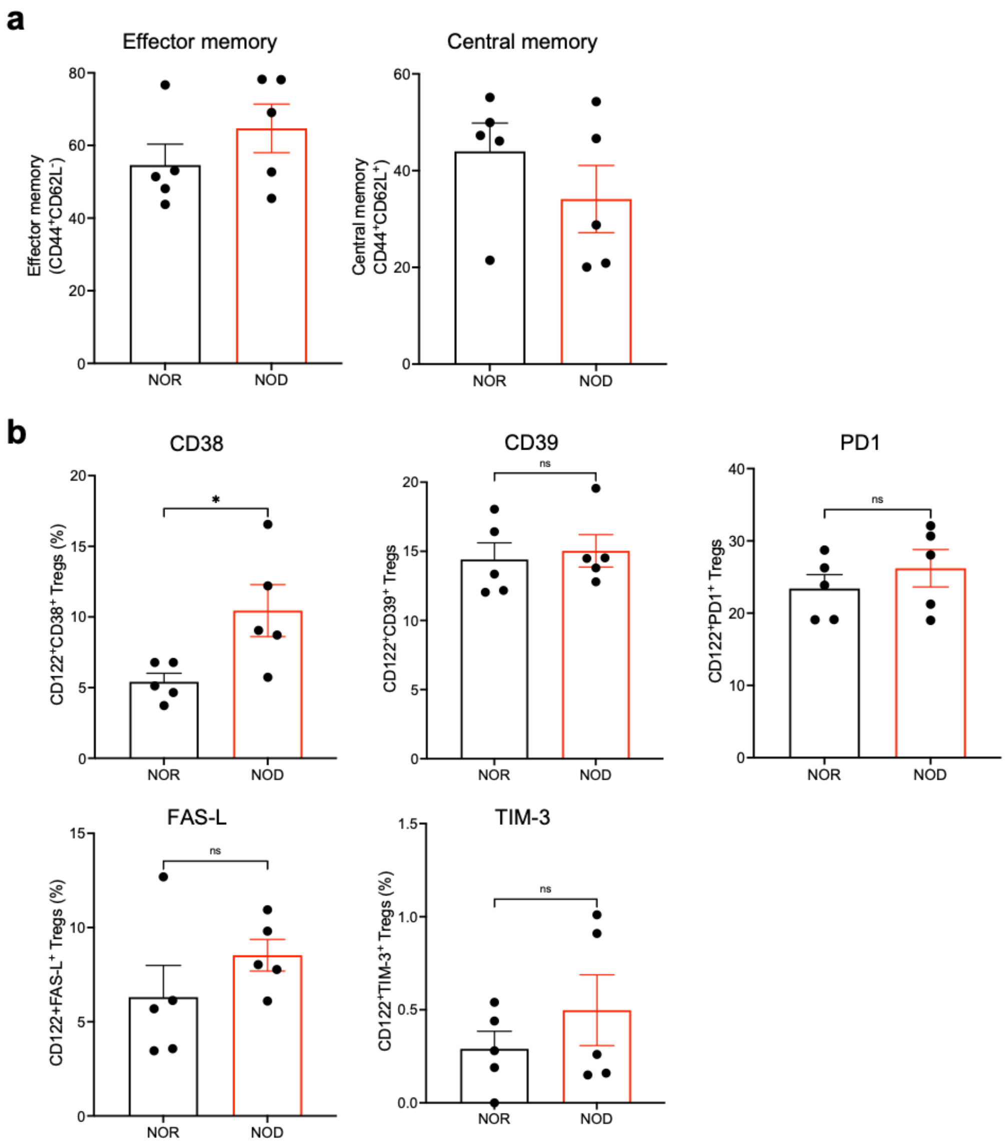

Fig S2. Phenotypic characterization of CD8$^+$CD122$^+$ Tregs from NOR and NOD mice. (**a**) Percentage of effector memory (CD44$^+$CD62L$^-$) Tregs and central memory (CD44$^+$CD62L$^+$) CD8$^+$CD122$^+$ Tregs in NOR and NOD. (**b**) Expression of different surface markers in NOR and NOD CD8$^+$CD122$^+$ Tregs. *p<0.05. ns: not significant. FAS-L: Fas ligand, NOD: non-obese diabetic, NOR: non-obese diabetes-resistant, PD1: programmed cell-death protein 1, TIM-3: T-cell immunoglobulin and mucin-domain containing-3.

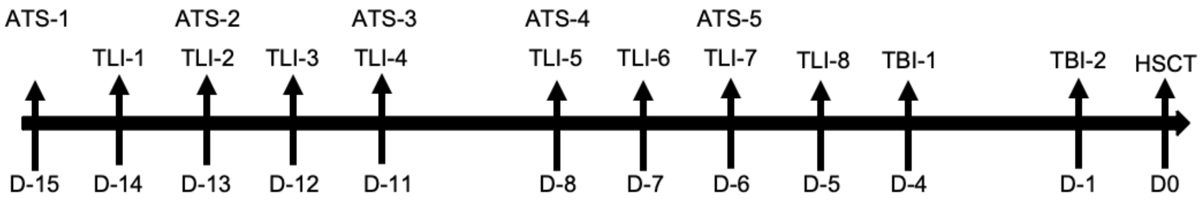

Fig S3. Details of the conditioning regimen used for HSCT. Prediabetic NOD mice were conditioned with 8 doses of total lymphoid irradiation (2.4 Gy each dose), two doses of total body irradiation (1.5 Gy each dose), and 5 doses of anti-thymocyte serum (50 μL each dose). TLI was given at day -14, -13, -12, -11, -8, -7, -6, and -5 prior to HSCT. TBI was given at day -4 and day -1 prior to HSCT. ATS was given at day -15, -13, -11, -8, and -7 prior to HSCT. A total of 50×10^6 whole bone marrow cells from C57BL/6 was injected into each mouse at day 0. ATS: antithymocyte serum, D: day, HSCT: hematopoietic stem cell transplantation, NOD: non-obese diabetic, TBI: total body irradiation, TLI: total lymphoid irradiation.

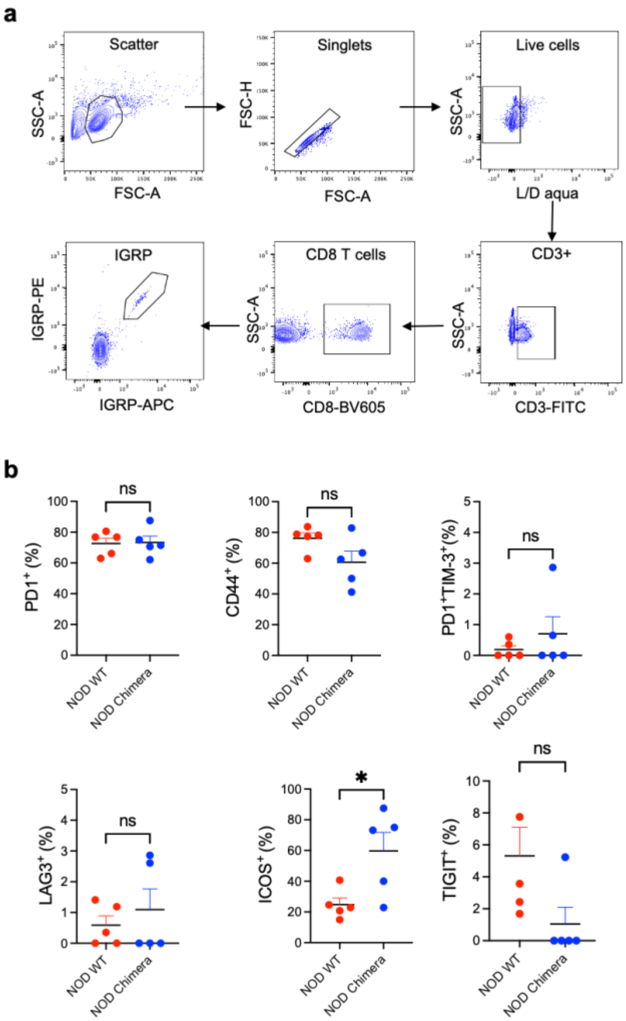

Fig S4. Detection and characterization of IGRP-reactive CD8^{+} T cells. (**a**) Gating hierarchy of IGRP-reactive CD8^{+} T cells in NOD mice. Dead cells were excluded by staining with Zombie Aqua™ Fixable Viability Kit. CD3^{+}CD8^{+} T cells that were double-positive for IGRP-APC and IGRP-PE tetramers were considered IGRP-reactive CD8^{+} T cells. (**b**) Expression of different surface markers in IGRP-reactive CD8^{+} T cells in NOD WT and NOD chimera. *p<0.05. ns: not significant. ICOS: inducible T-cell costimulator, IGRP: islet-specific glucose-6-phosphatase subunit-related protein, LAG3: lymphocyte activation gene 3, PD1: programmed cell-death protein 1, TIGIT: T cell immunoreceptor with Ig and ITIM domains, TIM-3: T-cell immunoglobulin and mucin-domain containing-3, WT: wildtype.

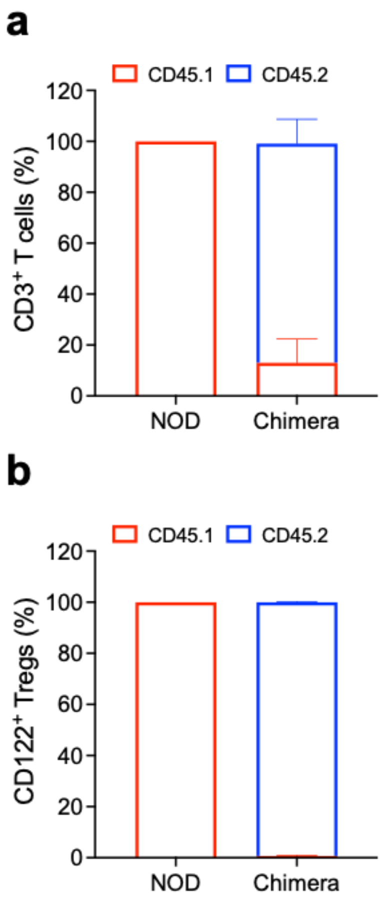

Fig S5. Donor $CD8^+CD122^+$ Tregs expand in the pancreas of chimeric NOD mice. NOD chimeras were euthanized at day 60 post-HSCT, pancreata were collected and digested using 1 mg/mL collagenase IV (Millipore Sigma) at 37°C for 10 min. Lymphocytes were isolated from digested pancreata by density gradient centrifugation using Ficoll-Paque™ PLUS (Cytiva). Isolated lymphocytes were stained with antibody against CD3, CD8, CD44, CD122, CD45.1, and CD45.2. Flow cytometry was used to analyze T cells in pancreata. T cells isolated from pancreata of NOD mice were used as controls. (**a**) Donor and host composition of $CD3^+$ T cells in the pancreas of chimeric mice. (**b**) Donor and host composition of $CD8^+CD122^+$ Tregs in pancreas of chimeric mice. Donor-derived cells were identified by CD45.2 and host-derived cells were identified by CD45.1 staining. d-$CD122^+$ Tregs: d-$CD8^+CD122^+$ Tregs, NOD: non-obese diabetic.

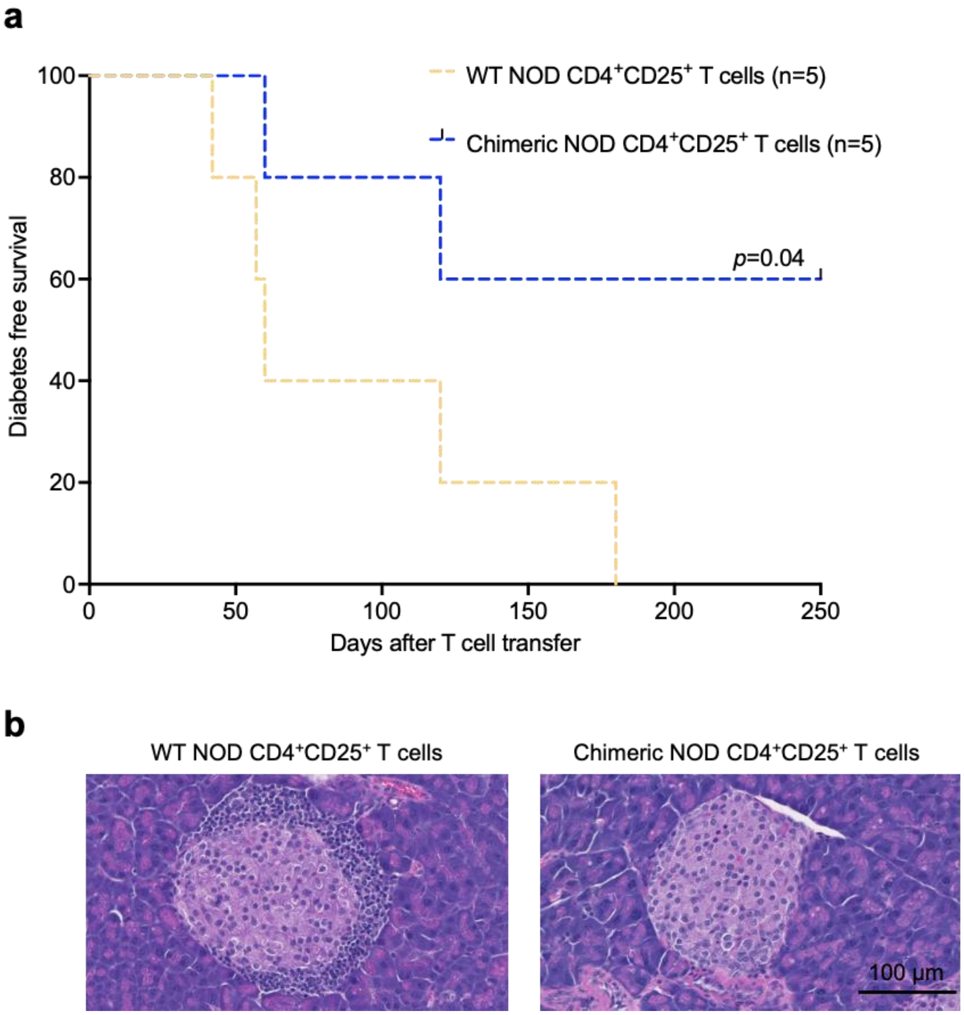

Fig S6. Host $CD25^+$ T cells isolated from NOD chimera prevent diabetes onset. NOD T cells (2×10^6 per mouse) and $CD4^+CD25^+$ T cells from WT NOD or $CD45.1^+CD4^+CD25^+$ T cells from chimeric NOD mice (0.5×10^6 per mouse) were simultaneously injected into NRG mice. Non-fasting blood glucose levels of the recipients were then monitored weekly for diabetes induction and mice were considered diabetic when two consecutive blood glucose readings were over 300 mg/dL. (**a**) Kaplan-Meier curve showing diabetes free survival. (**b**) Hematoxylin and eosin staining of pancreas section. Magnification: 40×, Scale bar: 100 μm. NOD: non-obese diabetic, WT: wildtype.

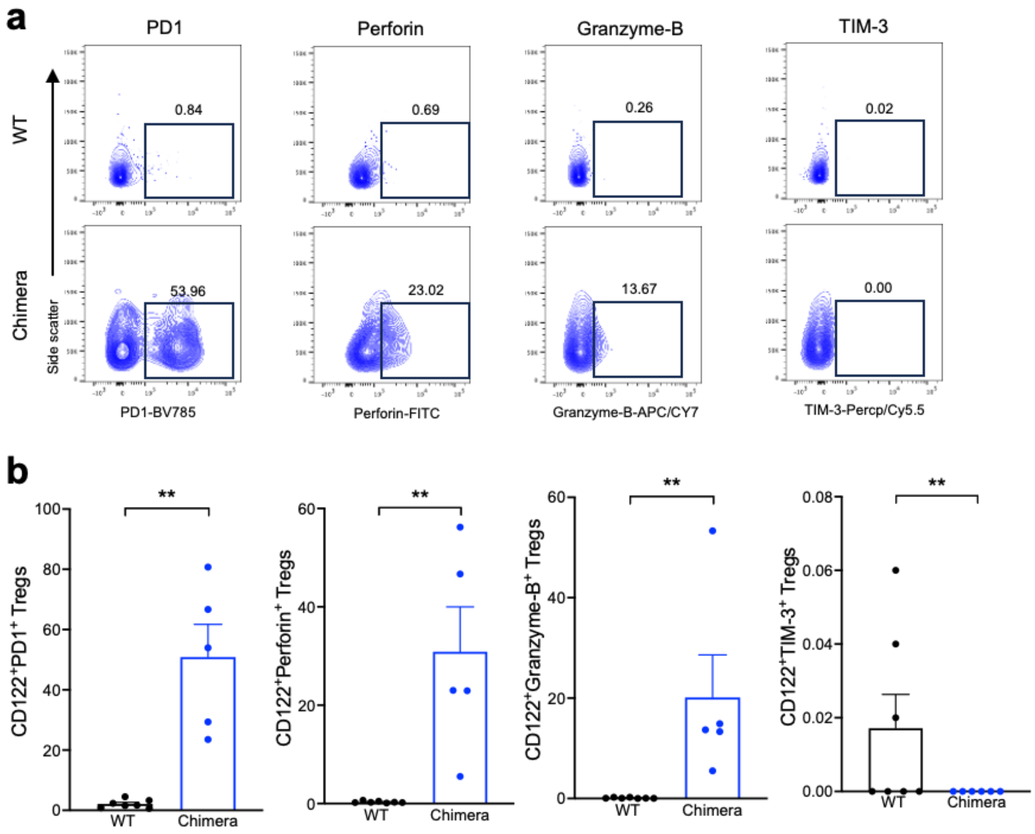

Fig S7. Phenotype of bone marrow-derived d-CD8$^+$CD122$^+$ Tregs. NOD chimeras were euthanized at day 60 post-HSCT and bone marrow cells were collected by flushing the femur with PBS. Age-matched WT C57BL/6 mice were used as controls. Dead cells were excluded by staining with Zombie Aqua™ Fixable Viability Kit. Cells were stained using antibodies against CD45.1, CD45.2, CD3, CD4, CD8, CD44, CD122, PD1, Perforin, Granzyme-B, and TIM-3. (**a**) Representative flow cytometry plots showing expression of PD1, Perforin, Granzyme-B, and TIM-3 in bone marrow-derived d-CD8$^+$CD122$^+$ Tregs from NOD chimeras and WT C57BL/6 mice. (**b**) Quantification of the percentage of PD1, Perforin, and Granzyme-B, and TIM-3 positive cells among CD8$^+$CD122$^+$ Tregs. **$p<0.01$. d-CD122$^+$ Tregs: d-CD8$^+$CD122$^+$ Tregs, PD1: programmed cell-death protein 1, TIM-3: T-cell immunoglobulin and mucin-domain containing-3, WT: wildtype.

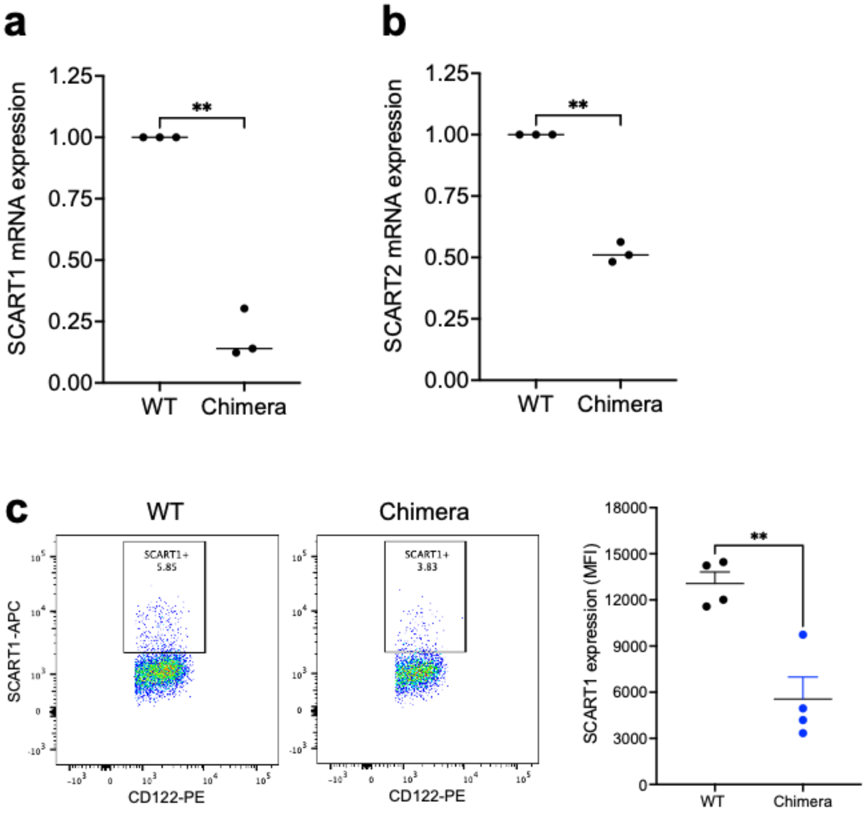

Fig S8. Donor-derived CD8$^+$CD122$^+$ Tregs have reduced expression Scart1 and Scart2. (**a-b**) qRT-PCR analysis was performed to verify the expression of the mRNA levels of Scart1 and Scart2. (**a**) mRNA expression of Scart1 in CD8$^+$CD122$^+$ Tregs. (**b**) mRNA expression of Scart2 in CD8$^+$CD122$^+$ Tregs. (**c-d**) Flow cytometric validation of Scart1 protein expression in CD8$^+$CD122$^+$ Tregs. Primary antibody against Scart1/CD163L1 (Novus Biologicals) was conjugated with FITC using a FlexAble FITC Plus Antibody Labeling Kit (Proteintech), following manufacturer's instructions. Lymphocytes from WT C57BL/6 and chimeric mice were then stained with antibodies against CD45.1, CD45.2, CD3, CD4, CD8, CD44, CD122, and CD163L1-FITC. (**c**) Representative flow cytometry plots showing expression of Scart1 protein in CD8$^+$CD122$^+$ Tregs. (**d**) Quantification of mean fluorescence intensity of Scart1 in CD8$^+$CD122$^+$ Tregs. ***$p<0.01$. SCART: scavenger receptor family member expressed on T cells, WT: wildtype.

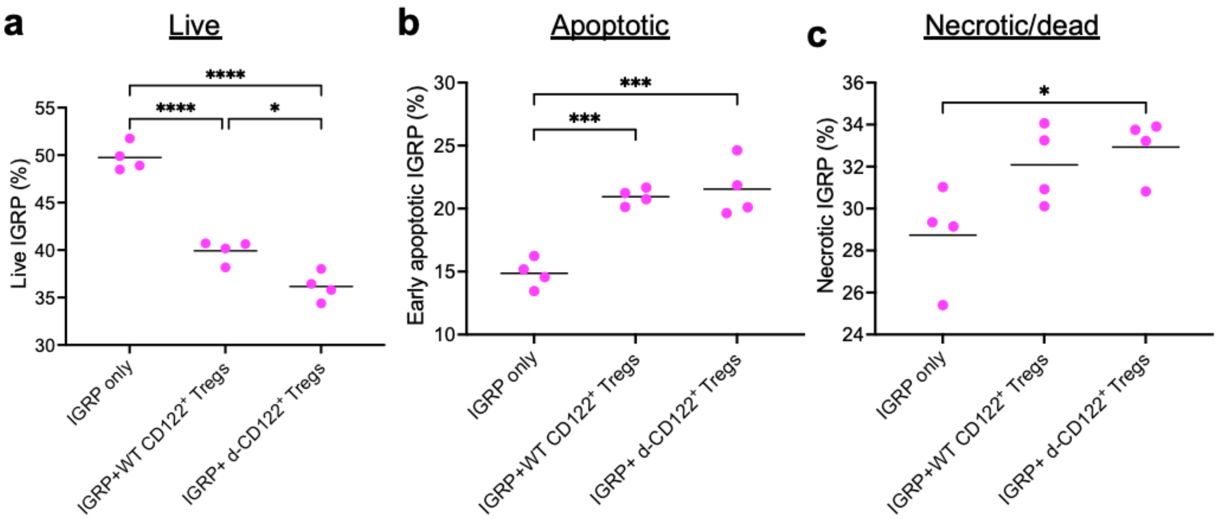

Fig S9. Donor-derived $CD8^+CD122^+$ Tregs have higher cytotoxicity effects towards IGRP-reactive $CD8^+$ T cells. T cells were isolated form NOD mice, stained with IGRP-PE tetramer, and magnetically enriched for IGRP-reactive $CD8^+$ T cells using anti-PE microbeads. Then, 1×10^5 IGRP-enriched NOD T cells were co-cultured with 5×10^4 $CD8^+CD122^+$ Tregs for 72 h. IGRP-reactive $CD8^+$ T cell killing was assessed by NucView®488 and RedDot™ 2 Apoptosis and Necrosis Kit (Biotium) using manufacturer's protocol. Percentages of live (NucView® 488^- RedDot™ 2^-), early apoptotic (NucView® 488^+), and late apoptotic/necrotic (NucView® 488^+RedDot™ 2^+) cells were assessed among the $CD45.1^+CD8^+IGRP^+$ fraction. (**a**) Percentage of live cells. (**c**) Percentage of early apoptotic cells. (c) Percentage of late apoptotic/necrotic cells. *p<0.05, ***p<0.001, ****p<0.0001. d-$CD122^+$ Tregs: d-$CD8^+CD122^+$ Tregs, IGRP: islet-specific glucose-6-phosphatase subunit-related protein, WT: wildtype.

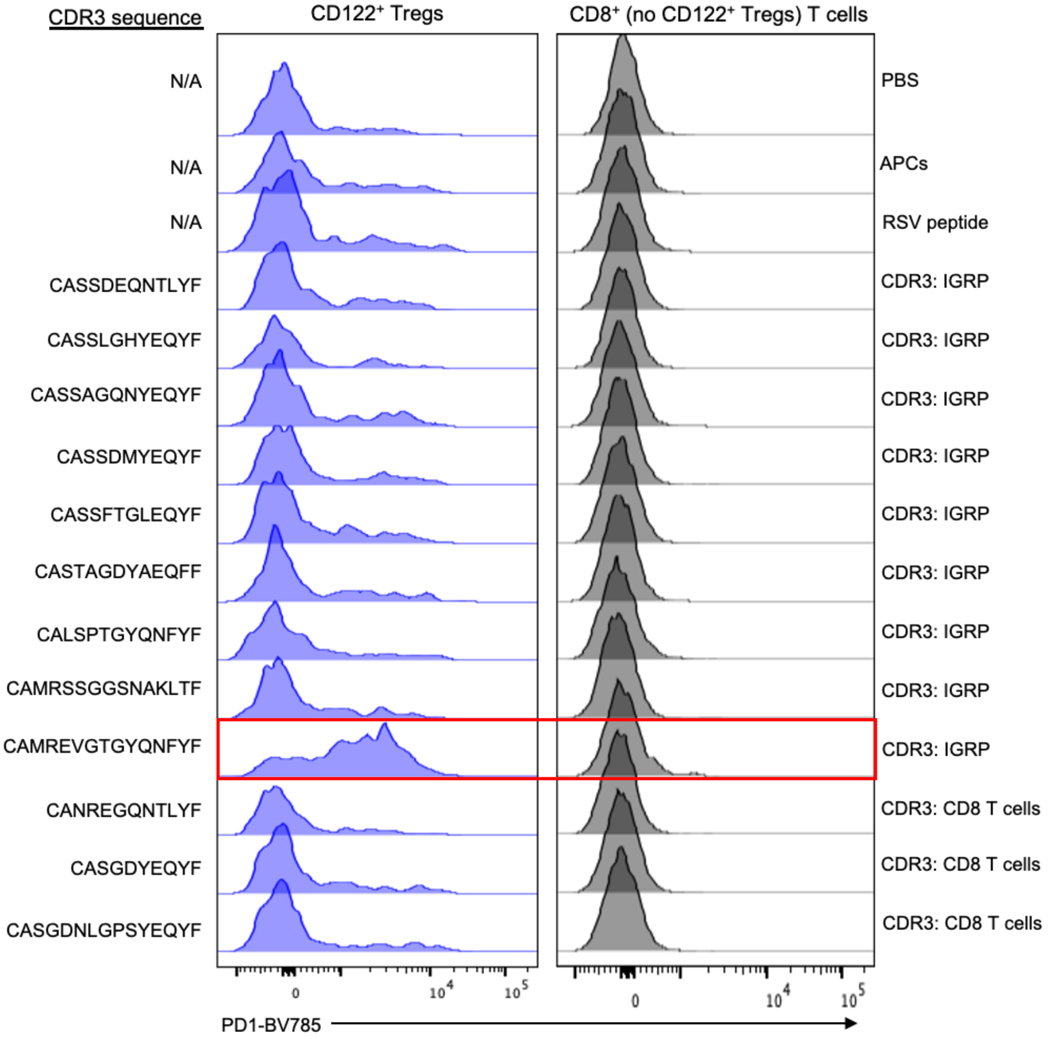

Fig S10. Screening of CDR3 peptides using activation assay. Nine CDR3 peptide sequences derived from IGRP-reactive CD8$^+$ T cells and three CDR3 peptide sequences derived from NOD CD8$^+$ bulk T cells were added separately in the co-culture of d-CD8$^+$CD122$^+$ Tregs or CD122-depleted d-CD8$^+$ T cells, and CD11b$^+$ monocytes as APCs for 48 h. RSV peptide (sequence: SYIGSINNI) was used as an irrelevant control. All peptides were used at a concentration of 20 μM. Representative histograms showing expression of PD1 in CD8$^+$CD122$^+$ Tregs (left) and CD122-depleted d-CD8$^+$ T (right) are shown. d-CD122$^+$ Tregs: CDR3: complementarity-determining region-3, d-CD122$^+$ Tregs: d-CD8$^+$CD122$^+$ Tregs, IGRP: islet-specific glucose-6-phosphatase subunit-related protein, N/A: not applicable, WT: wildtype.

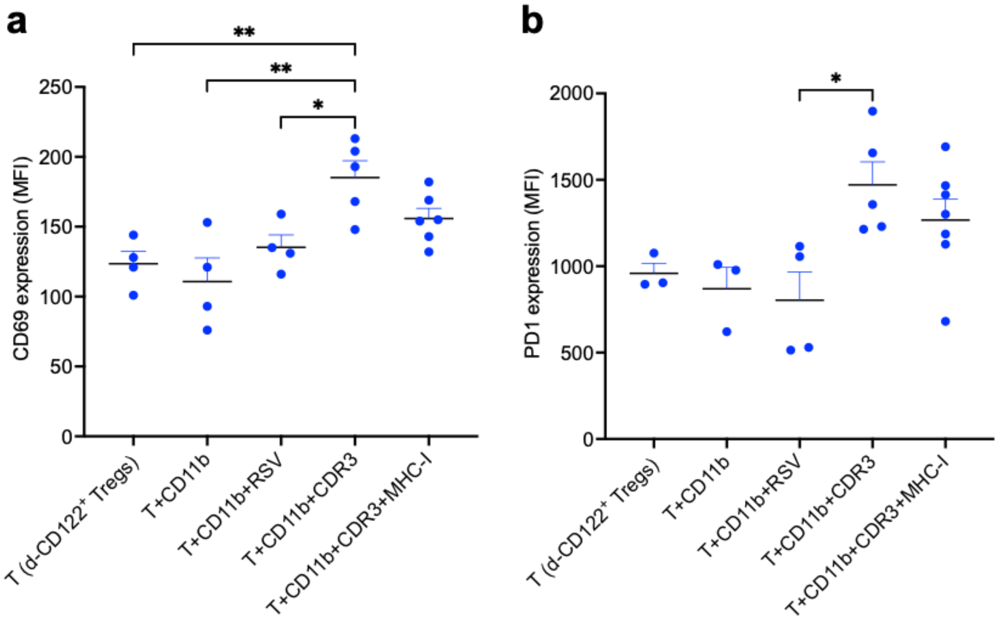

Fig S11. MHC-I blockade abrogated CDR3 peptide-mediated activation of d-CD8^{+}CD122^{+} Tregs. Pan MHC-I blocking antibody (10 μg/mL, BioXcell Cat no. BE0077) was added into coculture of d-CD8^{+}CD122^{+} Tregs and CD11b^{+} monocytes, and 20 μM of CDR3 peptide. Mean fluorescence intensity of CD69 and PD1 expression was quantified. (**a**) Expression of CD69. (**b**) Expression of PD1. *p<0.05, **p<0.01. CDR3: complementarity-determining region-3, d-CD122^{+} Tregs: d-CD8^{+}CD122^{+} Tregs, MFI: mean fluorescence intensity, MHC-I: major histocompatibility complex-I, PD1: programmed cell-death protein 1.

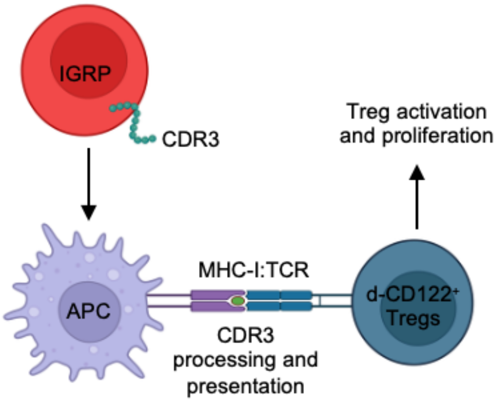

Fig S12. Proposed mechanism of IGRP-reactive CD8$^+$ T cell killing by d-CD8$^+$CD122$^+$ Tregs *in vitro*. The IGRP-reactive CD8$^+$ T cell-derived CDR3 peptides are processed by APCs and presented to d-CD8$^+$CD122$^+$ Tregs in the context of MHC-I. This leads to activation and proliferation of the d-CD8$^+$CD122$^+$ Tregs *in vitro*. APC: antigen presenting cell, CDR3: complementarity-determining region-3, d-CD122$^+$ Tregs: d-CD8$^+$CD122$^+$ Tregs, IGRP: islet-specific glucose-6-phosphatase subunit-related protein, TCR: T cell receptor.

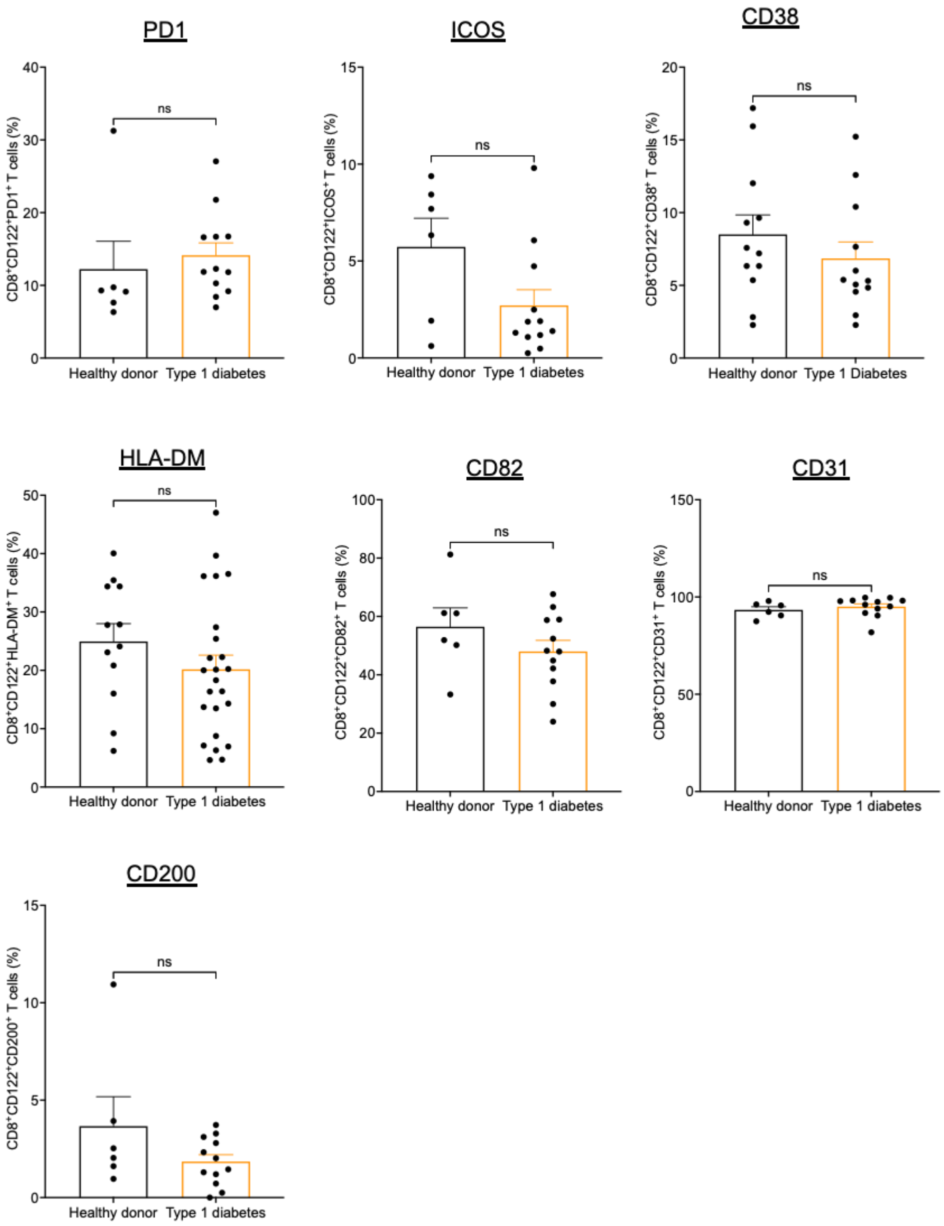

Fig S13. Phenotype of CD8^{+}CD122^{+} Tregs in individuals with T1D. PBMC samples from T1D and HD controls were stained with antibodies against PD1, ICOS, CD38, HLA-DM, CD82, CD31, and CD200. No statistically significant difference was observed in the expression of the surface markers among the CD8^{+}CD122^{+} Tregs in T1D and HD controls. ns: not significant. ICOS: inducible T cell stimulator, HLA-DM: human leukocyte antigen-DM, PD1: programmed cell-death protein 1.

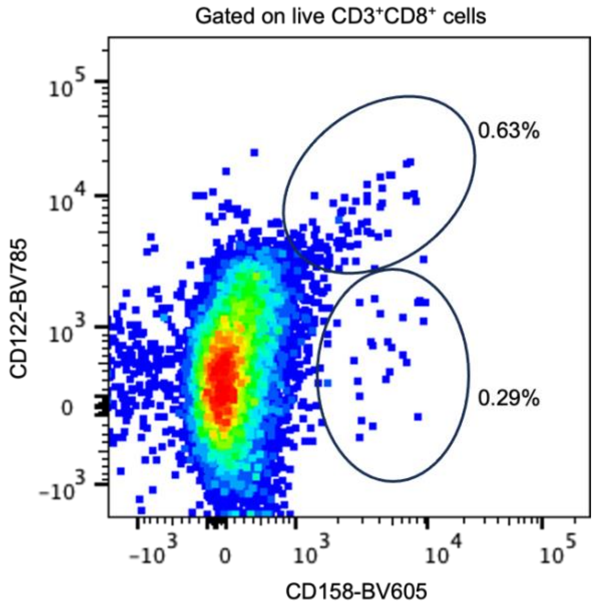

Fig S14. Representative flow cytometry plot showing overlapping phenotypic characteristics between $CD8^{+}CD122^{+}$ Tregs and $CD8^{+}CD158^{+}$ Tregs in human sample.

Table S1. Demographics of human samples used for Bulk RNA sequencing

SN	Group	Sex	Age (years)	Date of T1D onset	Blood draw
1	Healthy	unknown	unknown	N/A	2025/04/09
2	Healthy	unknown	unknown	N/A	2025/04/09
3	Healthy	unknown	unknown	N/A	2025/04/09
4	Healthy	unknown	unknown	N/A	2025/04/30
5	Healthy	unknown	unknown	N/A	2025/04/30
6	Healthy	unknown	unknown	N/A	2025/04/30
7	T1D	M	17	2024	2025/06/06
8	T1D	F	78	2008	2025/06/13
9	T1D	M	43	1999	2025/07/30
10	T1D	M	43	2024	2025/07/30
11	T1D	F	73	1973	2025/08/27
